# Zinc: a metallic shield against cardiac inflammation

**DOI:** 10.1093/mtomcs/mfag004

**Published:** 2026-02-26

**Authors:** Kemmoy Lattibeaudiere, Shelly McFarlane, Marvin Reid, Irina Korichneva, John H Beattie

**Affiliations:** School of Natural and Applied Sciences, University of Technology, Kingston, Jamaica; Caribbean Institute for Health Research (CAIHR), The University of the West Indies, Mona, Jamaica; Caribbean Institute for Health Research (CAIHR), The University of the West Indies, Mona, Jamaica; Université de Picardie Jules Verne, Department of Pharmacology, 7517 MP3CV, CURS, Amiens, France; The Rowett Institute, University of Aberdeen, Aberdeen, United Kingdom

## Abstract

Zinc (Zn) is a trace element essential for the function of over 10% of the human proteome, yet the average adult body contains only about two grams. Despite its trace status, Zn plays an indispensable role in immune regulation, inflammation control, and redox signalling. Low Zn status is associated with impaired immune function and increased oxidative stress—factors that critically contribute to the pathogenesis of cardiac inflammatory diseases (CIDs), including myocarditis and pericarditis. These conditions are rising in incidence globally, particularly in younger adults, and are linked to viral infections, autoimmune triggers, and post-vaccination inflammatory responses. Zn not only protects cysteine thiol groups from oxidation but also acts as a redox-sensitive secondary messenger via the “Redox Zinc Switch” mechanism—a key process in modulating cellular responses to oxidative stress. In the cardiovascular system, Zn influences antioxidant defence, cytokine regulation, and membrane repair pathways, including cellular responses that are regulated by protein kinase C and metallothioneins. Emerging evidence supports Zn supplementation as a strategy to mitigate myocardial inflammation, reduce cardiac remodelling, and improve outcomes in oxidative stress driven heart diseases. This review synthesizes current knowledge on Zn’s biochemical, immunological, and therapeutic roles in cardiac inflammation. We argue that maintaining optimal Zn levels through diet or supplementation represents a promising, accessible intervention to reduce the burden of CIDs and improve cardiovascular resilience in at-risk populations.

## Graphical Abstract

**Graphical Abstract:**
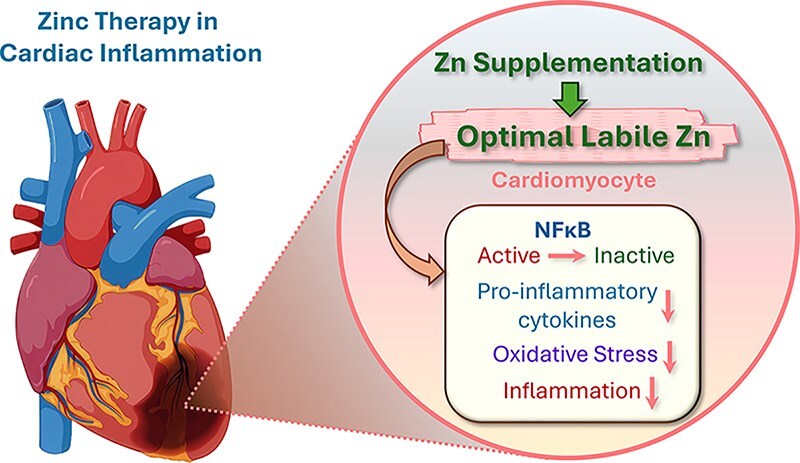
Zinc therapy in cardiac inflammation

## Introduction

The chemical characteristics of zinc (Zn) have been utilized extensively in cell biology to fulfil structural, enzymatic and signalling functions. This includes the modulation of several biochemical and signalling pathways associated with inflammatory responses, oxidative stress, and lipid and glucose oxidation [1–4]). A negative correlation between the level of serum Zn and the percentage body fat and glycaemic status within individuals has been observed [2, 5, 6]. Additionally, the role of Zn in the synthesis, storage and release of the glucose regulatory hormone, insulin, is well-documented [7, 8]. Consequently, Zn supplements may offer a therapeutic benefit to type 2 diabetics with the potential to reduce symptoms and associated complications [7, 8]. Other diverse physiological roles reported for Zn include promotion of cellular growth and division, healing of damaged tissues, DNA synthesis, reduced mortality in children due to diarrhoea and pneumonia and alleviating symptoms of microbial infections, such as the common cold, chronic hepatitis C, and leprosy [9]. The importance underscores the need for sufficient Zn intake where primary dietary sources include oysters, legumes, seafood, and beef; however, Zn bioavailability may be significantly reduced by inhibitors such as phytates present in many plant-based foods [10–-12].

The cellular demand for Zn varies depending on the strength of Zn-protein interactions and the local redox environment. As Zn status declines, the complexity and severity of deficiency-related symptoms increase. Acute Zn deficiency characterized by growth retardation, alopecia, and skin lesions is uncommon with otherwise balanced diets and is typically observed in cases of severe nutritional deprivation (e.g. Prasad’s study of Egyptian dwarfs [13]) or genetic disorders affecting Zn absorption, such as acrodermatitis enteropathica [14]. In contrast, marginal Zn deficiency, more prevalent globally, can result from chronically low intake and often presents with non-specific clinical features, including impaired immune function. These effects are frequently compounded by other nutritional or environmental factors.

Zn plays a critical role in immune regulation, influencing the development, maturation, and function of both innate and adaptive immune cells [3]. Deficiency is associated with impaired lymphocyte signalling, increased susceptibility to infections, and reduced life expectancy, particularly in vulnerable populations such as the elderly [4]. In addition to its immunomodulatory functions, Zn is a key regulator of inflammatory responses [4, 15–-19]. Although inflammation is a natural protective response to injury or infection, chronic or unresolved inflammation can exacerbate numerous disease processes. It has been implicated in the pathophysiology of asthma, cardiovascular diseases, diabetes mellitus, and rheumatoid arthritis [20].

Importantly, Zn may have specific relevance in the context of cardiac inflammation. Conditions such as myocarditis and pericarditis, which involve inflammation of the heart muscle and surrounding pericardial tissues, respectively, have been associated with sudden cardiac death in 5%–22% of young athletes [21]. These conditions are often underdiagnosed and may result from viral infections, exposure to environmental toxins, or idiopathic causes [22]. Furthermore, inflammatory cardiac disorders have been implicated in sudden infant death syndrome [23] and reported in patients with SARS-CoV-2 (COVID-19) infection [24, 25].

Although the literature, particularly regarding specific biomarkers of Zn status in cardiac inflammation, is limited, available evidence points to an association between Zn deficiencies and cardiac pathologies. In their work on Zn status, Ripa et al. [26] concluded that a deficit of Zn is among the causes of structural cardiomyocyte impairments. Similarly, Rosenblum et al. [27] describes a case study in which oral Zn therapy restored symptoms of heart failure in a patient with low Zn status. Furthermore, during chronic inflammation, plasma Zn deficiency may reduce antioxidant enzyme levels, leading to increased myocardial apoptosis and necrosis.

Given the capacity of Zn to influence both immune and cardiac function [4, 16], these observations have fuelled growing interest in the therapeutic and prophylactic potential of this micronutrient to influence the initiation and progression of cardiac inflammatory diseases (CIDs). This review, therefore, examines the physiological significance of Zn in inflammation which could mitigate the impact of inflammatory cardiac conditions.

## The inflammatory response

In response to pathogens, damaged cells, and other harmful stimuli, the immune system typically responds by stimulating inflammation [18, 28]. This process is a defence mechanism that promotes healing, whilst aiding in the elimination of invading microbes or toxins [18, 29]. However, in some instances, acute inflammation progresses to chronic stages and may contribute to the pathogenesis of several chronic conditions including rheumatoid arthritis, atherosclerosis, diabetes, and obesity [29–31]. Furthermore, adipose tissues secrete inflammatory cytokines, therefore, obesity can systemically increase tissue exposure to pro-inflammatory conditions [32].

Despite the nature of the stimulus, inflammatory responses follow a consistent sequence: damage recognition, activation of pathways, release of inflammatory markers, and immune cell recruitment [18]. Microbial components known as pathogen-associated molecular patterns (PAMPs) activate pattern recognition receptors (PRRs), such as toll-like receptors (TLRs), which recognize both microbial threats and danger-associated molecular patterns (DAMPs) from injured tissues [18, 33].

Activation of TLRs results in a biochemical cascade promoting translocation of transcription factors, including activator protein-1, nuclear factor kappa B (NF-κB) and mitogen-activated protein kinase (MAPK), [18, 19]. Among these, NF-κB is extensively studied due to its regulatory role in inflammation, immunity, and apoptosis [34–-36]. It is ubiquitously expressed and can be activated by pathogens, cytokines, and enzymes [18]. An unregulated or poorly coordinated inflammatory pathway plays a role in the development of varying life-threatening illnesses [29]. For many of these illnesses, dysregulation of NF-κB has been implicated. Under physiological conditions, the transcription factor is maintained in an inactive form by binding of the cytoplasmic inhibitor protein IκB. Activation of PRRs triggers the activation of the enzyme IκB kinase, ultimately allowing NF-κB to bind to its promoter sites, resulting in the transcription of pro-inflammatory mediators [19, 37].

Other transcription factors similarly drive immune responses by regulating the expression of cytokines, growth factors, and hormones [18]. These cytokines, including interleukins (IL-4, IL-6, IL-8, IL-10, IL-11), tumour necrosis factor-alpha (TNF-α), interferon-gamma (IFN-γ), and transforming growth factor-beta (TGF-β), are released primarily by monocytes and macrophages [18, 19, 37]. They mediate pro- and anti-inflammatory effects, thus serving a central role in the inflammatory response. For example, IL-4, IL-10 and IL-11 act as major anti-inflammatory cytokines, and in their absence, inflammatory responses increase by ∼50% in neutrophils within surrounding tissues [38].

Elevated levels of several cytokines are used in the prognosis of inflammation related illnesses, such as heart failure, cardiovascular diseases, diabetes mellitus, and cancer. Furthermore, elevation of oxidative stress markers may also serve as indicators of inflammation [30, 31]. The implications can be severe, suggesting that tight regulation of the immune response is paramount in reducing the risk of chronic inflammation. In many viral infections, including COVID-19, pro-inflammatory cytokines such as TNF-α and IL-1 are released within tissues, thus recruiting leukocytes to those healthy tissues, leading to an inflammatory response [39]. These cytokines may persist for up to 80 d, resulting in the development of associated complications [40–42]. Therefore, it is relevant to investigate key factors regulating the inflammatory response, which in turn has therapeutic value for attenuating inflammation-associated diseases.

## Zinc modulation of inflammation

During the inflammatory response, Zn is a central modulator through several mechanisms. Animal model research shows that a decrease in the intra-cellular Zn pool reduces the clearance of bacterial, viral and fungal infections [43]. This could be explained by a dysregulation in the synthesis and secretion of IL-2, IL-12, and IFN-γ [44]. These cytokines are required for maximum macrophage phagocytic activity against invading pathogens. Consequently, Zn plays a major role in signalling the immune system for the clearance of pathogens. Other studies have established a direct correlation between serum Zn levels and immunity. For example, Raqib et al. [45] reported on the beneficial role of 14 d Zn supplementation in children diagnosed with shigellosis, an infection caused by *Shigella*. The supplement resulted in a significant elevation in the proliferation of lymphocytes. Further research confirmed that Zn effectively activates the innate immune response and regulates inflammation in response to various pathogens in children, highlighting its key biochemical properties [19]. The immune response assisted with the elimination of several pathogens including the common cold, *Mycobacterium tuberculosis*, and leptospirosis. Similarly, Zn supplements reduced infections and chronic inflammation in geriatric patients through modulation of inflammatory cytokines. Furthermore, the importance of Zn homeostasis in the regulation of inflammation using cell lines, human promyelocytic leukaemia cell line (HL-60), human monocytic leukaemia cell line (THP-1) and human aortic endothelial cells (HAEC) has been reported. Incubation of the cell lines with oxLDL in the presence of Zn resulted in a significant depression of pro-inflammatory cytokines through a decrease in the expression of the transcription factor, NF-κB [17, 46]. In summary, the research on the importance of the trace metal in the immune response and regulation of inflammation is extensive and encompasses the use of animal models, human cell lines and human subjects. The findings strongly support the contention that Zn is a key beneficial factor in maintaining optimal immunity and in the regulation of inflammation.

**Figure 1:**
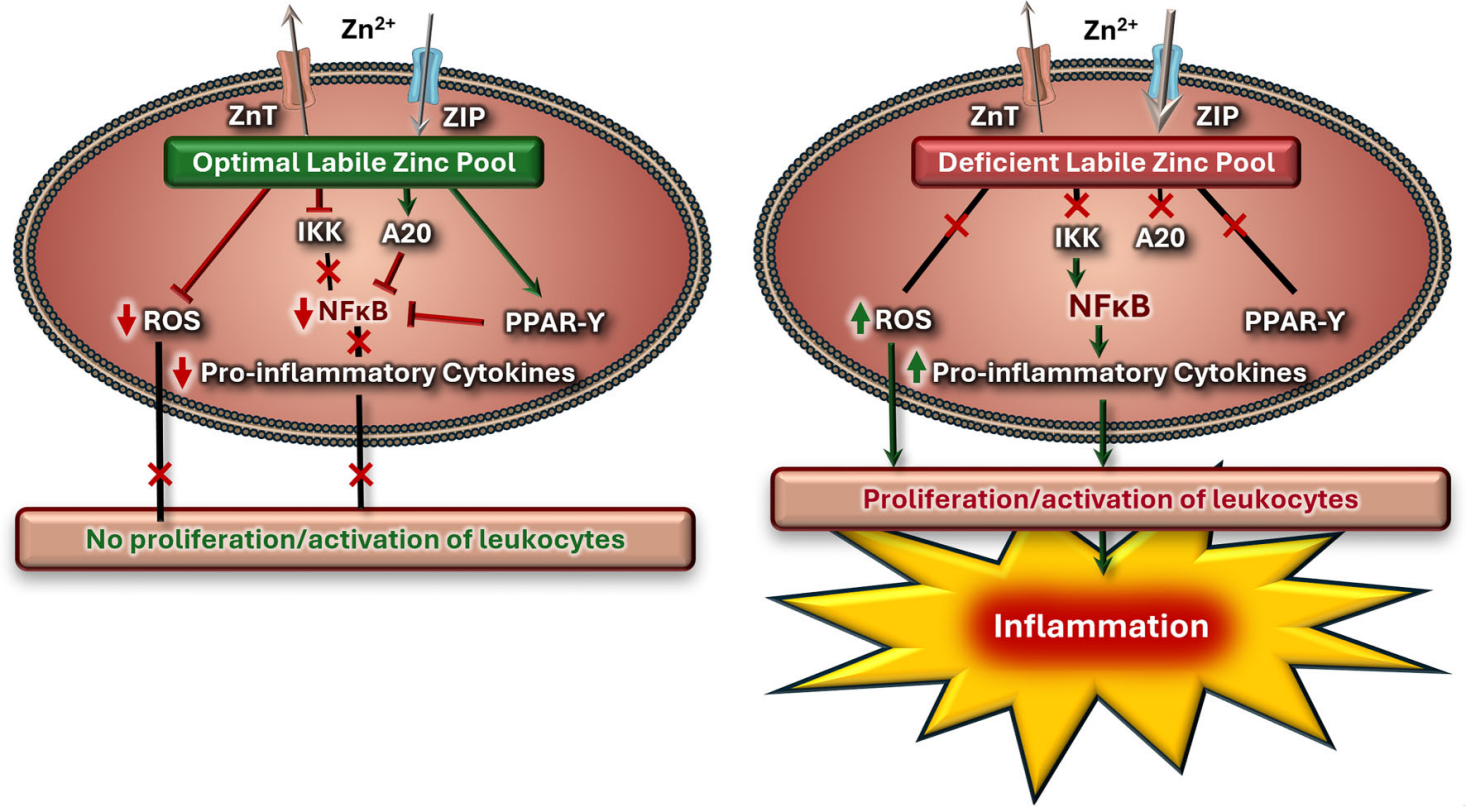
the role of zn status in modulating inflammation. the level of cellular zn is tightly regulated by the zn transporters, ZIP and znt. cellular labile zn binds to A20 proteins thus down-regulating NF-κB, the transcription factor required for the expression of several pro-inflammatory cytokines. additionally, labile zn inhibits IKK (IκB kinase) thus preventing the phosphorylation of iκb. zn also increases the expression of the transcription factor, PPAR- γ, inactivating the action of NF-κB, thus reducing inflammatory responses. furthermore, zn suppresses ROS, consequently reducing tissue damages and therefore reducing tissue inflammation due to oxidative damage. zn: zinc, znt: zinc transporter, ZIP: zrt-/irt like protein, ROS: reactive oxygen species, PPAR-γ: peroxisome proliferator-activated receptor gamma, NF-κB; nuclear factor kappa B.

The mechanisms of inflammation modulation have been studied at length and, to date, have not been completely elucidated and some remain controversial. The literature supports Zn playing a pivotal role in the suppression of inflammation (Fig. 1). Zn deficiency is highly correlated with the over expression of pro-inflammatory cytokines such as IL-1β, IL-6 and TNF-α [17–19, 47]. These are all under the control of NF-κB, indicating that available Zn is essential for the inhibition of the transcription factor. There are several mechanisms that have been proposed to explain the interaction between the two. Of these, the activation of a Zn finger protein, A20, has gained the most attention. The protein is described as a major anti-inflammatory factor in the regulation of inflammatory responses. Das et al. [48] reported on the spontaneous development of autoinflammatory diseases in mice following targeted cell-specific deletion of the A20 gene, thus highlighting the importance of the protein in modulating inflammation. Furthermore, A20 negatively regulates tumour necrosis factor receptor (TNFR) and the TLR-initiated NF-κB signalling pathway [2, 17, 19]. The anti-inflammatory protein, A20 de-ubiquitinates receptor interacting protein 1 (RIP1), thus preventing the interaction with essential activators of NF-κB [49]. The process regulates the stability and activity of RIP1, ultimately reducing the possibility of binding and therefore, the transcriptions of pro-inflammatory cytokines [17]. Interestingly, the expression of A20 is regulated via a negative feedback mechanism with NF-κB, allowing for constant regulation of the inflammation process [47]. Another factor that regulates the expression of A20 is the divalent cation, Zn (Fig. 1) [19]. The process of A20 DNA transcription is Zn-dependent, and Zn supplements reduce the expression of major pro-inflammatory cytokines through an increase in A20, which reduces the cytokine transcription. Moreover, Zn is required for the stability and activation of the protein, further underscoring the need for Zn in the modulation of inflammation [17].

Zn may reduce the expression of NF-κB through the increased expression of another transcription factor, peroxisome proliferator-activated receptor-γ (PPAR-γ). PPAR-γ is pivotal for insulin sensitization, glucose metabolism and adipocyte differentiation. However, PPAR-γ has been implicated in terminating the activation of NF-κB [50, 51]. This is achieved through the ubiquitin ligation of NF-κB/Rel A (a member of the NF-κB family) that results in the degradation of, and hence termination of Rel A, causing in a decrease in pro-inflammatory cytokines and therefore, in inflammation. The importance of Zn in the activation of PPAR-γ was highlighted using chelating agents, which demonstrated that there was a consequent decrease in the expression of PPAR-γ, with an elevation in NF-κB and IL-6 [50]. Furthermore, Zn increases the level of cGMP through an inhibition of phosphodiesterase enzymes [19]. Elevation of the nucleotide activates protein kinase A (PKA), which phosphorylates and inhibits the expression of genes under the influence of NF-κB [52]. The literature is, therefore, highly supportive of the anti-inflammatory property of Zn through the indirect modulation of NF-κB, which plays a key role in the regulation of inflammation. Consequently, Zn supplements may offer a therapeutic approach in mitigating chronic inflammation and inflammation-coupled complications. However, it is worth examining further mechanisms that may be involved in Zn regulation in the inflammatory process.

Zn may be involved in the interaction of TLRs. As reported, these are a group of receptors that recognize PAMPs and activate transcription factors essential for the inflammatory response. In the monocytes, the activation of TLR4 results in the phosphorylation and activation of MAPK and NF-κB [53]. Physiological Zn levels are essential in preventing the dephosphorylation by enzymes such as protein tyrosine phosphatase and cyclic nucleotide phosphodiesterase [19, 53, 54]. This activates the expression of pro-inflammatory cytokines, and therefore inflammation. Paradoxically, Zn is required for the activation of the inflammatory response, but it also plays an inhibitory role in the dephosphorylation process and hence reduces inflammation [54].

Other mechanisms through which Zn exerts an effect on inflammation include apoptosis, on the synthesis and expression of pro-inflammatory cytokines, and on oxidative stress [19]. The latter demonstrates the association between the intra-cellular Zn status and the production and reduction of reactive species [2, 19]. This scheme is detailed below in Fig. 1. The physiological effects of Zn in regulating inflammatory responses are well-established, and so Zn should therefore be considered as a therapeutic treatment in chronic inflammation. The therapeutic effects of Zn can be explained by an offset of complications linked to elevated inflammatory responses [2, 19]. Consequently, it is of value to examine some of the beneficial roles of Zn in chronic inflammation-related diseases, particularly CIDs.

## Zinc and oxidative stress

Oxidative stress occurs when the generation of reactive oxygen and nitrogen species (ROS/RNS) exceeds the capacity of cellular antioxidant defences, leading to macromolecular damage and impaired tissue function [30]. While moderate ROS levels participate in physiological signalling and innate immune defence, chronic, or excessive oxidative burden contributes to inflammation-related diseases such as atherosclerosis, diabetes, Alzheimer’s disease, and myocarditis [55].

Cells counteract oxidative stress through enzymatic antioxidants—superoxide dismutase (SOD), catalase, and glutathione reductase—and non-enzymatic molecules such as glutathione (GSH), ascorbic acid, and α-tocopherol [30, 56]. Among micronutrients, Zn plays a central role in maintaining redox balance. Although redox-inert, Zn influences oxidative status indirectly by stabilizing thiol groups, displacing redox-active metals (Fe²⁺, Cu²⁺) from catalytic sites, and serving as a structural or catalytic cofactor for antioxidant enzymes [2, 57].

**Figure 2:**
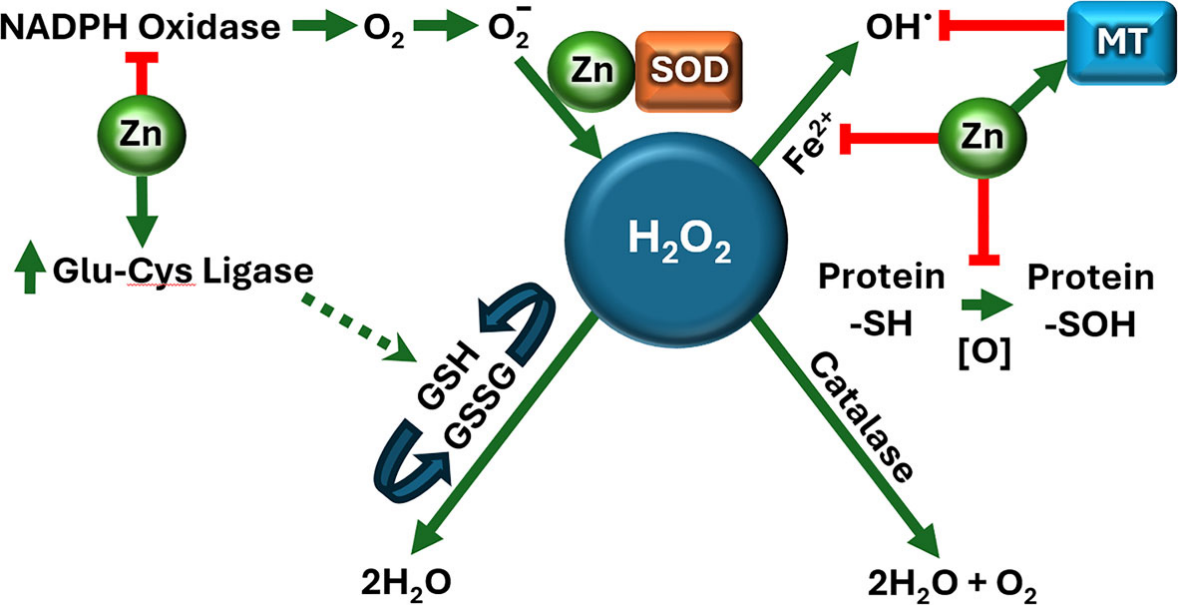
Mechanisms of action of Zn in reducing oxidative stress. The straight green arrows indicate stimulation of expression; the dotted green arrow indicates indirect, increased expression; red lines indicate inhibition or scavenging. NADPH oxidase produces O₂⁻, which is converted to H₂O₂ by Zn-dependent SOD. H₂O₂ can form hydroxyl radicals (•OH) via Fe²⁺-catalysed Fenton reactions. Labile Zn²⁺ displaces Fe²⁺, reducing •OH formation. Zn up-regulates glutamate-cysteine ligase (GCL) for glutathione (GSH) synthesis, while catalase reduces H₂O₂ accumulation. Zn also protects protein thiol (-SH) groups from oxidation. Abbreviations: NADPH oxidase = nicotinamide adenine dinucleotide phosphate oxidase; O₂⁻ = superoxide; SOD = superoxide dismutase; Fe²⁺ = ferrous ion; •OH = hydroxyl radical; MT = metallothionein; GCL = glutamate-cysteine ligase; GSH = reduced glutathione; GSSG = oxidized glutathione; Protein-SH = thiol group; [O] = oxidation; Protein-SOH = oxidized thiol group.

A principal antioxidant function of Zn is its role as a cofactor for Cu/Zn-SOD, which catalyzes the dismutation of the superoxide anion (O₂⁻) to hydrogen peroxide (H₂O₂), thereby preventing excessive superoxide accumulation (Fig. 2) [58]. Zn deficiency markedly reduces SOD activity, increasing susceptibility to oxidative damage [59, 60]. Additionally, intra-cellular Zn competes with transition metals for binding sites on membranes and metalloproteins, limiting the Fenton-type generation of hydroxyl radicals (•OH) from H₂O₂ [59]. Physiological Zn concentrations thus inhibit the formation of ROS, including •OH [2].

Zn also modulates antioxidant defence by regulating glutathione synthesis. The rate-limiting enzyme, GCL, contains a metal-response-element (MRE) promoter through which Zn enhances its expression [59, 61]. Conversely, Zn deficiency decreases GCL expression and GSH levels, elevating oxidative stress. Zn further acts as an inhibitor of NADPH oxidase, a major source of ROS during inflammatory responses [2, 59]. Through these combined effects, Zn suppresses oxidative damage and mitigates chronic inflammation.

Beyond these indirect antioxidant mechanisms, Zn directly protects cellular macromolecules by binding to cysteine thiol groups and preventing their irreversible oxidation [2]. Many proteins contain thiol groups essential for catalytic or structural integrity; these are particularly sensitive to oxidation. Zn binding stabilizes such thiols, maintaining enzymatic activity and protein conformation. Metallothioneins (MTs)—cysteine-rich Zn-binding proteins—serve as intra-cellular Zn buffers and ROS scavengers [62, 63]. Zn-mediated thiol protection has been implicated in reducing protein oxidation associated with ageing and age-related diseases such as macular degeneration and cardiovascular disease [62, 64–66]. Long-term Zn supplementation decreases the formation of oxidized thiols (—SOH,—SO₂H,—SO₃H) and may thus preserve tissue function [62, 66, 67].

Through these multifaceted mechanisms, Zn acts as a key modulator of oxidative stress, maintaining redox homeostasis and protecting cellular structures. The protective role of

Zn is based on its unique chemical nature, which is redox-neutral but capable of changing protein redox state when bound to thiols. Zn-cysteine interactions that confer thiol protection underpin a dynamic regulatory process termed the “Redox Zinc Switch”, in which redox-dependent Zn release or rebinding modulates protein function.

## The redox zinc switch

Building on the antioxidant and thiol-protective roles of Zn described above, changes in the cellular redox state can also modulate Zn binding dynamically within proteins, giving rise to the concept of the *Redox Zinc Switch* [68]. This mechanism provides an additional layer of control, where cysteine residues that coordinate Zn become targets of reversible oxidation, thereby coupling oxidative cues to protein structure and function.

Most frequently, cysteine-bound Zn acts as a stabilizing structural component of Zn-finger domains, maintaining protein conformation and mediating protein—protein or protein—DNA interactions. Under reducing conditions, the chelated Zn ions are tightly coordinated with cysteine thiols and histidines, as seen in transcription factors and several cytosolic signalling proteins [69]. Oxidative conditions; however, weaken cysteine—Zn affinity, leading to Zn release from these coordination sites and triggering conformational and functional changes.

Such Zn-dependent redox control was first characterized for the bacterial protein HSP33 [70]. In human physiology, analogous redox control of Zn signalling has been described in multiple signalling pathways, but the Redox Zinc Switch activation of a protein was first described for the activation of protein kinase C (PKC) [70], with the delta and epsilon isoforms being the key regulators of myocardial signalling under oxidative stress.

The redox response of signalling proteins is not limited to PKC isoforms. A similar principle applies to Raf kinase, a component of the MAPK cascade [71], the GTPase-activating proteins Vav, non-protein-kinase phorbol-ester receptors chimaerins, and the transcription activator Keap1 [72, 73]. MAPK-pathway enzymes and the Keap1/Nrf2 axis are activated during preconditioning; as a result, mild hypoxia confers resistance to ischemia/reperfusion-induced oxidative stress in the heart. Another example of Redox Zinc-Switch control relevant to myocardial function is MG53, a tripartite-motif (TRIM) family protein that is essential for membrane-repair processes. MG53 acts as an oxidation sensor that recruits intra-cellular vesicles to the injury site for membrane patch formation [82]. MG53 ablation results in defective membrane repair and progressive pathological changes in skeletal and cardiac muscle. Domain-homology analysis shows that MG53 contains two Zn-binding domains—a RING-finger and a B-box motif—and that Zn binding is indispensable for membrane-injury repair.

The redox response thus serves both adaptive and protective purposes. Zn release during the first stage of the redox response serves as an adaptation to stress, and it further develops into protection through enabling cells to reset redox status and inducing antioxidant defences, a preconditioning mechanism known as *sublethal stress*. This prepares tissues for more severe oxidative challenges. Zn-dependent signalling proteins such as PKC, PI3K/Akt, MAPK-pathway enzymes, and Keap1/Nrf2 are activated during such preconditioning, rendering the heart more resistant to ischemia/reperfusion-induced oxidative stress.

**Figure 3:**
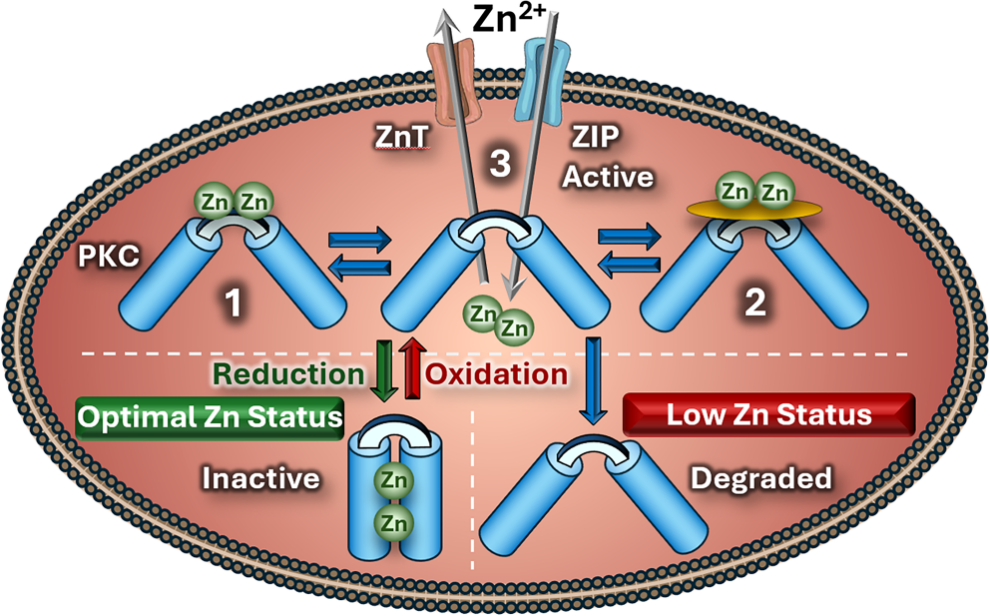
hypothetical model of zn-dependent conformational changes of PKC under oxidation/reduction. in the adaptive redox response, oxidation of cysteines triggers zn release and unfolds the regulatory domain of the kinase into an active conformation, with three possible fates for zn: (1) binding to a non-cysteine kinase domain, (2) association with a partner protein, or (3) removal through znt transporters under prolonged stress. the proximity of zn ions is physiologically important for regaining the inactive conformation, which is compromised under zn deficiency, leading to protein degradation.

Figure 3 presents different scenarios that would impact on cellular proteins based on Zn status. In normal physiology, the Redox Zinc Switch responds to reversible oxidation/reduction cycles with Zn serving as a linchpin. Prolonged stress triggers export of Zn excess in cytoplasm to prevent toxicity. The intra-cellular available Zn concentration decreases and, consequently, signalling proteins cannot refold properly and undergo degradation, possibly leading to the early stage of cell senescence and death.

In conditions of Zn deficiency, the functions of Zn-dependent proteins—and particularly the adaptive responses mediated through the Redox Zinc Switch—are compromised. We therefore hypothesize that Zn release during redox activation is a transient event that does not lead to lasting Zn depletion. Zn ions likely remain in proximity to the proteins governed by the Redox Zinc Switch, possibly sequestered by non-cysteine-rich acceptors such as His-rich SLC30A (ZnT) family members. Upon restoration of reducing conditions, Zn is re-captured by Zn-finger proteins to re-establish their resting conformations. Under severe or prolonged oxidative stress, when cysteines remain oxidized, Zn demand diminishes and excess Zn is secreted through efflux transporters. Consistent with this mechanism, elevated Zn levels have been detected in perfusates of ischemic hearts immediately upon reperfusion following total occlusion.

## The role of MT in zinc homeostasis

The biochemical form, localisation, and homeostasis of cellular Zn significantly influence the onset and progression of various diseases. As a result, Zn levels and speciation are tightly regulated through processes such as absorption, sequestration, and efflux across cell membranes [74]. Bioavailable Zn derived from Zn-rich food, such as red meat, is primarily absorbed through the duodenal mucosa (enterocytes) by Zn transporters, whereas the primary route for Zn excretion is via secretions from the exocrine pancreas back into the gut. ZIP4, located on the enterocyte apical membrane, facilitates Zn uptake [75, 76], while ZnT1 on the basolateral membrane assists in moving Zn from enterocytes into the portal circulation [3]. Though the full mechanisms and energy sources for Zn transport are not fully understood, other transporters, including ZnT-5 and ZIP5, are also implicated [3, 76]. Notably, ZIP4 is considered the primary mucosal influx transporter, as *ZIP4* gene mutations are linked to the severe Zn deficiency observed in acrodermatitis enteropathica [77].

High levels of intra-cellular labile Zn have toxic effects on the function of the cells, and as such must be regulated. This is achieved through Zn expulsion from cells, containment within vesicles called zincosomes, as well as through binding to high Zn affinity proteins called MTs. These are low molecular weight proteins that are rich in cysteine residues and as such can bind up to seven Zn ions to one molecule of the protein [78–80]. MT-1 and MT-2 isoforms contain 61 amino acids, with the thiol groups of 21 cysteine residues forming seven tetrathiolate clusters with Zn^2+^ ions in a two-domain stable structure. The high affinity of Zn binding in tetrathiolate clusters and the domain protein conformation resists metal dissociation under normal cytoplasmic reducing conditions. However, the dissociation constant of each Zn cluster varies between 10^−8 ^M and 10^−12 ^M, and metals in the beta domain are more labile. Study of the protein structure reveals that there needs to be a conformational change to facilitate the dissociation of the metal ions. The lowering of pH and/or a change in redox status favouring oxidation, promotes the release of Zn. Thus, by binding and releasing Zn within cells, MTs fulfil their major role as a Zn buffer. Maret’s group have drawn a distinction between steady state intra-cellular Zn buffering and non-steady state Zn muffling, to explain the complex spatial and temporal dynamics of free Zn management permitting its signalling role and protecting against its toxicity [63]. The cellular concentration of MT is adjusted to meet the buffering and muffling demand through activation of the Zn finger metal-responsive transcription factor-1 MTF-1 in the presence of excess labile Zn and expression promotion of Zn regulatory proteins, including MTs, through MRE promoters [80]. Elevated MT levels increase the binding capacity for Zn and reduce the cellular levels of free/labile Zn ions. Other factors that promote MT gene expression include other transition metals such as Cu or Cd and ROS. In the case of Cd, the metal ions have a higher affinity for thiol ligands than Zn, and they therefore displace Zn from MT, resulting in elevated levels of labile Zn, activation of MTF-1 and an increase in MT gene transcription [81].

Free Zn ions dissociate from the thiol groups of MTs when intra-cellular Zn has been depleted. This process plays a pivotal role in maintaining intra-cellular Zn concentrations, which are required for different biochemical processes [82]. The release of Zn through oxidation of MT thiols promotes the neutralisation of free radical species through mechanisms already described in this review, and a return to redox homeostasis. Although MT is not quantitatively a potent antioxidant compared to e.g. glutathione, Zn-MT complexes interact with, and reduce ROS, such as superoxide and hydroxyl species [83]. The combined influence of Zn release and direct antioxidant properties help protect DNA against damage from ROS and promote replication of DNA by supplying Zn to DNA polymerases and other Zn requiring processes in the nucleus of a dividing cell. This may explain why MT migrates from the cytoplasm to the nucleus during the G1 to S phase of cell division [84]. The cellular Zn content is also modulated to regulate the synthesis of proteins, inflammatory responses, lipid and glucose oxidation, and wound healing, among other processes. MT knockout mice have low plasma Zn concentrations, ultimately retarding bone formation, increasing cardiac remodelling through oxidative stress and an impaired immune response to foreign antigens [85, 86]. These findings underscore the importance of MT in regulating bioavailable Zn for cellular processes and is therefore important for maintaining Zn homeostatic control.

## Zinc status and cardiac ischaemia and perfusion

A chronically elevated state of oxidative stress is associated with cardiovascular diseases, neurological diseases, and metabolic diseases, among others. The myocardium and pericardium are also susceptible to the deleterious effects of reactive species, and there is a strong positive correlation between oxidative stress and heart failure [87, 88]. The oxygen supply to the cardiac tissues is almost twice that of other tissues, resulting in a potentially higher rate of reactive species (ROS/RNS) generation. Principal sources of these agents within the heart are the mitochondrial electron transport chain, NADPH oxidase, xanthine oxidase, and NOs. As described above, antioxidants scavenge ROS/RNS, but an elevation of reactive species may overwhelm the heart’s endogenous antioxidants.

When free radical generation is increased, there is a depression of adenosine triphosphate (ATP) and an elevation of cardiac remodelling [89]. ROS also affects the electrophysiology and contractile mechanism of the heart through protein modifications, including various ion-gated channels [90]. These are both associated with the pathophysiology of varying heart conditions, including cardiac ischaemia. Under such conditions, there is a reduction in blood flow (and therefore oxygen) to the cardiac muscles, which promotes oxidative stress within the heart tissues [91]. An increase in oxidative stress following cardiac ischaemia and reperfusion may contribute to the pathogenesis of several cardiovascular complications [88]. Acknowledging the involvement of reactive species in these injuries, attention has focussed on the potential therapeutic value of targeting oxidative stress as a preventative measure for ameliorating cardiac ischaemia/reperfusion and related complications [92, 93]. In fact, clinical trials using varying antioxidants such as resveratrol, n-acetylcysteine and flavonoids all showed reduced oxidative stress and offered cardiac protection against ROS/RNS associated injuries in ischaemia [93].

Given the beneficial effects of Zn in mitigating oxidative stress, it may have a protective role against cardiac ischaemia/reperfusion injuries and associated complications. Yu et al. [94] reported on the correlation between impaired Zn homeostasis and ischaemic cardiomyopathy, atherosclerosis and cardiac ischaemia with reperfusion. Further studies underscored the importance of Zn for cardiac function, not only as an antioxidant but also as a secondary messenger within the heart and reduces the occurrence of cardiac apoptosis [95–97]. Moreover, the effects of Zn on oxidative stress have been detailed above and, through these mechanisms, Zn reduces the deleterious effects of reactive species generated during and after cardiac ischaemic/reperfusion. There is considerable evidence to support this, and Zn deficiency is related to increased cardiac remodelling and cardiac dysfunction due to oxidative stress [98, 99]. Moreover, there is much published support for the therapeutic use of Zn supplements to reduce cardiac diseases including atherosclerosis of the coronary arteries, myocardial infarction, and cardiac ischaemia [100]. The depletion of Zn, due to cardiac ischaemia and reperfusion, facilitates cardiac apoptosis, and explains the elevated cardiac dysfunction following ischaemia. This highlights the protective role Zn serves in ameliorating heart injuries due to ischaemia/reperfusion. Loss of Zn from cardiomyocytes following ischaemia is detectable in animal models by reperfusion effluent analysis (see Morand et al., this volume), but is not easy to determine in the whole-body circulation of human patients. Carbonic anhydrase (CA) II and IV, both of which bind a large proportion of myocardial Zn, are abundant in the heart and we speculate that they may be released into circulation during ischemia. Using CA II or IV as a biomarker for heart derived Zn in patients after myocardial infarction might be an indirect way to monitor losses of Zn from the heart muscle.

Zn is also beneficial due to its role as a modulator and secondary messenger in other biochemical processes. Since cardiac ischaemia depletes myocardium Zn levels, this may aggravate the incidence of cardiac injuries through disturbed redox balance [101–103]. Zn supplementation may be of therapeutic value for cardiac ischaemia [101, 103]. When administered just before reperfusion, Zn pyrithione reduces oxidative stress and preserves cardiac cells in cultured neonatal rat cardiomyocytes [101] and improved myocardial recovery up to 100% in male Sprague–Dawley rats [103], underscoring the cardioprotective role of Zn. Oral administration of Zn supplements in animal models reduced cardiac injuries which resulted from cardiac ischaemia/reperfusion, indicating that a disruption of Zn homeostasis is involved in the pathophysiology of these diseases [104, 105]. One mechanism by which this is achieved is through the Zn modulation of PKC, which is involved in the signal transduction and contraction of the heart [102, 106]. PKC isoforms play a central role in the signalling cascades triggered by oxidative stress imposed by I/R. The Redox Zinc Switch activates these enzymes through release of Zn from Zn fingers of the regulatory domains. While appearing controversial, the role of PKC in the I/R depends on responses of specific isoforms. As such, both PKC delta and PKC epsilon are activated by I/R. Prolonged oxidative stress will lead to degradation of these proteins because in the condition of decrease intrac-ellular Zn they would be able to refold to proper conformation.

To overcome intra-cellular Zn deficiency and recharge the myocardial tissue with Zn using perfused rat heart model we bypassed Zn transport through membrane Zip proteins and ion channels by using Zn ionophore pyrithione in the reperfusion solution. We observed that PKC isoforms were protected from degradation with intra-cellular Zn supplementation [103]. Interestingly, Zn reduces the mitochondrial translocation of the PKC delta and increases migration to cytoskeletal structures instead, consequently reducing deleterious effects in cardiomyocytes [107]. Overall, these data suggests that pharmacological substances with the properties of Zn ionophores may serve as cardioprotectants in the conditions of oxidative stress in the diseased and ageing hearts.

The role of Zn in reducing the risks of cardiovascular diseases such as coronary artery atherosclerosis, myocardial infarction, heart failure and cardiac ischaemia is well documented. A depletion of free/labile intra-cellular levels of Zn is highly associated with an increased risk of cardiac diseases through elevated oxidative stress. This is even more noticeable with the ischaemic/reperfusion injuries, where there is an increase of ROS/RNS and decreased levels of intra-cellular Zn. Supplements of Zn offset some of the injuries through its antioxidant effects that include the positive modulation of SOD expression, the protection of thiol groups within proteins, inhibition of the prooxidant enzyme NADPH oxidase as well as reducing the formation of other ROS and RNS [2, 58]. The pharmacological property of such supplements reduces cardiac remodelling and therefore may serve a potential role in the treatment of heart diseases.

## Potential impact of zinc on myocarditis and pericarditis development

Myocarditis is an inflammatory disease of the myocardium and is predominantly caused by viral infections, including adenovirus, parvovirus B19, and SARS-CoV-2 which causes the coronavirus disease of 2019 (COVID-19) [108–110]. However, inflammation in the myocardium can be triggered by various stimuli beyond viral infections, including autoimmune disorders, drug reactions, and bacterial pathogens [103, 108, 109]. Myocarditis can lead to death through heart failure, but early treatment including anti-viral drugs, antibiotics and anti-inflammatory drugs (e.g. corticosteroids) usually results in recovery, albeit with a risk of lasting damage to the myocardium [110, 111]. Clinical prognosis varies widely, ranging from full recovery to chronic heart failure or sudden cardiac death, depending on the severity and timeliness of treatment. Prognostic assessment often incorporates biomarkers such as N-terminal pro-B-type natriuretic peptide (NT-proBNP) [112] and C-reactive protein (CRP), which correlate with the degree of inflammation [113]. The prevalence varies, with epidemiological studies reporting the number of cases being highly underdiagnosed, but estimating the global prevalence to ∼10.2–105.6 per 100 000 worldwide [109]. Over the last five years, there has been a marked increase in the incidence of the inflammatory disease, largely attributed to the COVID-19 pandemic. This increase is reported to exceed 10-fold since the COVID-19 pandemic and has been attributed to both the viral infection itself and administration of nucleic acid-based COVID-19 vaccines [114–117]. The common theme is exposure to the SARS-CoV-2 spike protein, but the heavy cell loading of the intact virus is predominantly in the respiratory tract, and less in the cardiovascular system.

Pericarditis, often seen as a comorbidity with myocarditis, is an inflammation of the pericardium, the cause of which is often unidentified. A variety of factors similar to those causing myocarditis has been indicated, such as viral infections, and treatments therefore attempt to reduce inflammation and address the cause of the disease, if known [118].

Many viruses are able to invade the cardiac tissues, thus triggering an inflammatory response. In COVID-19, a high viral load is correlated with the development of myocarditis through binding and entry via the angiotensin converting enzyme 2 (ACE-2) on cardiac myocytes [119]. The infection triggers a cytokine storm, including TNF-α and several interleukins, which causes recruitment of leukocytes within the cardiac tissues [120, 121]. The resulting ROS formation is one of the predominant driving factors involved in the deterioration of the myocardium in myocarditis. ROS are primarily produced by neutrophils and macrophages and serve as recruitment factors for additional leukocytes [122]. Furthermore, viral infections promote mitochondrial dysfunction which plays a critical role in the pathogenesis in myocarditis and pericarditis through further generation of ROS [123, 124]. Several infections viruses hijack the host mitochondrial machinery, thus impairing oxidative phosphorylation and resulting in the generation of ROS. These serve as major ROS, which further disrupt the functionality of several macromolecules and activate inflammatory pathways through NF-κB [124]. The ROS cause lipid peroxidation, protein oxidation, and DNA damage, leading to cell death and tissue fibrosis. Moreover, oxidative stress promotes cardiac apoptosis, remodelling and hypertrophy [125]. Ultimately, oxidative stress contributes to advanced stages of myocarditis through depression of arterial pressure, cell death, and exacerbated inflammation resulting from the elevation of leukocytes and inflammation-associated pathways, such as NF-κB [125].

A similar mechanism is involved in the pathogenesis of pericarditis. An increased oxidative stress within the pericardium leads to damaged endothelial and mesothelial cells, resulting in pericardial effusion and excessive collagen deposition. Consequently, this results in thickening and stiffening of the pericardium walls, a hallmark of pericarditis [126].

Current treatments for myocarditis and pericarditis aim to alleviate the underlying condition by administering antibiotic or antiviral agents in addition to reducing the immune response via immunosuppressive agents, immunoglobulins, anti-inflammatory agents and IL-1 antagonists [127, 128]. In advanced cases, treatments for heart failure including extracorporeal membrane oxygenation (ECMO) and anti-arrhythmic agents are employed [128]. Zn may offer a significant advantage due to its multi-channel mechanisms of action. Zn and MT possess antiviral properties, a particularly valuable trait given that viral infections are a leading cause of myocarditis. Furthermore, the clinical relevance of Zn in modulating inflammation is well established, not only via antioxidant pathways, but also through several other critical regulatory mechanisms, as described in this review. Non-viral aetiologies of myocarditis and pericarditis tend to be a consequence of other pathogens (such as parasitic and bacterial infections), or drug induced or autoimmune responses [129]. The molecular mechanism of the latter is poorly understood but may involve cardiac autoantibodies against autoantigens such as cardiac myosin, troponin and the M2 muscarinic receptor, among others [129]. Regardless of the trigger, the inflammatory responses appear to be similar and result in an overwhelming generation of oxidative stress. The literature highlights the pivotal role of oxidative stress in the pathology of myocarditis and pericarditis, regardless of its aetiology. Dependent on further study, targeting oxidative stress and its downstream effects may offer a promising therapeutic tool in inflammatory cardiac diseases.

## Discussion

Zinc, an essential trace element with antioxidant, anti-inflammatory, and immunomodulatory properties, plays a critical role in the maintenance of cardiovascular health, particularly in conditions like myocarditis and pericarditis [18]. These inflammatory heart diseases remain a therapeutic challenge, as current approaches lack depth in simultaneously targeting the core drivers such as oxidative stress, immune dysregulation, and viral or autoimmune triggers. Zn’s diverse biochemical roles directly target each of these pathological processes, making it a potentially comprehensive intervention strategy [17–19, 130].

The urgency for novel therapies has increased in the wake of COVID-19, where myocarditis and pericarditis have been observed in otherwise healthy individuals after SARS-CoV-2 infection and following mRNA vaccination [110, 116, 119]. The common factor between the virus and the vaccines is the presence or generation of the spike protein. In the case of the vaccines, the mRNA encoding bioactive spike protein is encapsulated in lipid nanoparticles and is systemically distributed to all accessible cells, including immune and heart cells [131, 132]. Once translated within these cells, the spike protein can be presented on the cell surface and has also been detected in blood plasma. For example, antibody-free spike protein was detected in the blood plasma of adolescents and young adults who developed myocarditis after mRNA vaccination, but not in plasma from vaccinated individuals with no subsequent myocarditis [133]. In some cases, spike protein may persist in plasma after vaccination for at least six months [134]. Circulating spike protein has the potential to adversely interact with ACE2 receptors expressed in various cell types, including cardiomyocytes [135]. Cardiac inflammation occurring after infection with the virus [116, 129] or COVID vaccination [115–118] is thought to be caused by the body’s intensified immune response and raised circulating cytokine levels. A therapeutic agent that can simultaneously regulate inflammation, protect against oxidative stress, and disrupt viral replication is critically needed—and Zn fulfils all three roles [19, 44, 49, 58–60, 136–138]. An online real-time meta-analysis of over 6100 studies suggests that Zn is indeed a cost-effective treatment for COVID-19, although this may depend on the Zn status of the individuals being studied [139].

Inflammation in myocarditis and pericarditis is characterized by elevated pro-inflammatory cytokines that drive leukocyte recruitment and tissue infiltration [140]. Zn modulates inflammation through the regulation of NF-κB, a central transcription factor in cytokine production and immune activation [17]. Zn deficiency results in elevated expression of pro-inflammatory cytokines such as IL-6 and TNF-α, which are also implicated in the pathology of myocarditis and pericarditis [54]. The Zn-dependent protein A20 is a potent inhibitor of NF-κB activation and appears critical in preventing uncontrolled inflammatory responses [17, 6]. An in vitro study with a HL-60, a promyelocytic cell line, demonstrated that Zn supplementation enhances A20 expression and stabilizes its activity, thereby dampening inflammatory pathways associated with cardiac injury [19, 140].

Furthermore, the redox-active nature of Zn is particularly important in mitigating oxidative stress, a key driver of cardiac inflammation. The myocardium, due to its high oxygen demand and dense mitochondrial content, is especially vulnerable to oxidative damage triggered by ROS/RNS released during myocardial stress or infection [141]. Zn deficiency exacerbates this damage by promoting immune cell dysregulation and leukocyte recruitment, contributing to chronic inflammation shown in an aged mouse model [47]. Zn supports antioxidant defences as a cofactor for SOD and by inducing glutathione synthesis, while also inhibiting NADPH oxidase and displacing redox-active metals like Fe²⁺ and Cu²⁺, thereby preventing further oxidative injury [2, 65, 142]. Both *in vitro* and *in vivo* models demonstrate that Zn supplementation reduces inflammation, highlighting its therapeutic potential in conditions such as myocarditis and pericarditis.

An intriguing concept outlined in this review is the Redox Zinc Switch; a redox-sensitive control mechanism whereby Zn dissociates from cysteine residues in Zn-finger proteins under oxidative conditions. This reversible release of Zn acts as both a redox sensor and signal transduction mechanism. Key cardiac signalling proteins, such as PKC, Raf, and MG53, are regulated by this mechanism. In particular, MG53 is critical for membrane repair in cardiac and skeletal muscle. Its Zn-binding domains are essential for its redox sensing and protective role during myocardial injury [80, 143]. The functional failure of these mechanisms under Zn-deficient conditions underscores the importance of Zn availability during oxidative stress [144]. Zn’s therapeutic relevance extends beyond its antioxidant capacity. It acts as a secondary messenger and modulator of cardioprotective signalling pathways, such as PI3K/Akt and PPAR-γ. Experimental models have shown that Zn supplementation can protect the heart from ischemia/reperfusion injury, reduce cardiac remodelling, and prevent mitochondrial apoptosis [86, 145]. Zn also appears to direct the subcellular localization of PKC isoforms, reducing their pro-apoptotic effects and preserving myocardial integrity [141].

In the context of SARS-CoV-2, the cardioprotective role of Zn extends beyond its immunomodulatory functions to include direct antiviral activity. Zn can inhibit RNA-dependent RNA polymerase, thereby disrupting viral replication [137, 138]. Oral administration of ionophores such as quercetin or epigallocatechin gallate may enhance dietary Zn uptake, overcoming the limitations of intestinal absorption at higher Zn doses [146, 147]. Likewise, reducing dietary phytate intake improves Zn bioavailability. When combined with its favourable safety profile and low cost, an optimized supplementation strategy that maximizes Zn absorption emerges as an attractive therapeutic adjunct for managing CIDs, particularly in individuals with suboptimal Zn status.

A persistent limitation in this field is the absence of a reliable biomarker for assessing individual Zn status, which hampers the design of adequately powered clinical and mechanistic studies. Plasma Zn concentration is a commonly used biomarker, but it lacks sufficient sensitivity to detect marginal deficiency at the level of individual patients, necessitating study populations in the hundreds to achieve the required statistical power. Consequently, much of the near-term progress will rely on animal and cellular models. For example, ischemia—reperfusion studies can help to elucidate how Zn modulates acute redox stress responses under the condition of viral burden.

Cardiomyocyte culture models remain valuable for dissecting Zn’s influence on cytokine signalling in the heart and on stress pathways. Computational modelling and artificial intelligence hold promise for identifying novel Zn ionophores, predicting drug—Zn interactions, and tailoring supplementation strategies to genetic and health profiles. Ultimately, a mechanistic framework for Zn-mediated cardio-protection will accelerate the translation of these findings into targeted clinical interventions, with the greatest potential impact in vulnerable populations such as the elderly and immunocompromised.

## Conclusions

Zinc is a multi-faceted micronutrient that regulates inflammation, oxidative stress, in some cases viral replication and also membrane repair—four central mechanisms in the pathogenesis of cardiac inflammation. Its role in modulating NF-κB, promoting antioxidant defences, and facilitating redox-dependent signalling suggests that adequate Zn status is essential for cardiovascular resilience. In clinical contexts marked by viral infection, autoimmune activation, or oxidative overload, Zn supplementation may offer prophylactic or therapeutic benefit. Future studies should aim to optimize Zn delivery strategies and identify biomarkers for Zn status to maximize patient benefit in cardiovascular inflammatory diseases.

## Data Availability

All research information and data used to write this review can be sourced in the cited references provided with the DOI or other website links.
